# Morphological Variation in the Human Mitral Valve Using Statistical Shape Modelling

**DOI:** 10.1007/s10439-026-04060-6

**Published:** 2026-03-19

**Authors:** Hyab Mehari Abraha, Chris Goddard, Rebecca Bryan, George Hyde-Linaker, Claire Conway

**Affiliations:** 1 Royal College of Surgeons in Ireland (RCSI), Dublin, Ireland; 2 Synopsys NE Ltd, Exeter, UK; 3 Trinity Centre for Biomedical Engineering, Trinity College Dublin, Dublin, Ireland

**Keywords:** Principal component analysis, Bicuspid valve, Geometric morphometrics, Coronary angiography

## Abstract

Statistical shape modelling (SSM) offers a robust framework for quantifying anatomical variability and constructing representative virtual patient cohorts of 3D anatomies that can be used as the foundation of biomechanical in silico clinical trials. In this study, we developed a SSM of the mitral valve using 72 contrast-enhanced computed tomography angiography (CTA) scans of the heart. Principal component analysis revealed dominant modes of shape variation that align with previously reported anatomical patterns in the literature, validating the model’s physiological relevance. The resulting shape model effectively captures the geometric diversity of the mitral valve without making any presuppositions about the importance of landmarks or linear measurements. Our results demonstrate the utility of SSMs in generating virtual patient populations from existing scan data. These findings support the integration of SSMs into computational modelling pipelines for preclinical testing, device design, and personalised medicine.

**Supplementary Information:**

The online version contains supplementary material available at https://doi.org/10.1007/s10439-026-04060-6.

## Introduction

Mitral regurgitation (MR) affects more than 24 million people globally and is a leading cause of morbidity and mortality [1]. MR is characterised by the retrograde flow of blood from the left ventricle to the left atrium during systolic ventricular ejection, imposing an increased volume load on the heart. If left untreated, it can progress to atrial fibrillation, pulmonary hypertension, and ultimately heart failure [2]. Although surgical mitral valve repair or replacement remains the gold standard treatment for MR, a subset of patients are deemed inoperable owing to age, comorbidities, or anatomical complexity [3].

Transcatheter mitral valve replacement (TMVR) has emerged as a minimally invasive alternative for high-risk patients [4–8], yet its clinical implementation remains limited. To date, three TMVR devices are approved for use in the European Union (EU)—Edwards Lifesciences SAPIEN3 [9], Abbott Tendyne [10, 11] and the HighLife Medical TMVR device [12]—and two approved for use in the US, the Abbott Tendyne and Edwards Sapien M3 [9]. However, unlike transcatheter aortic valve replacement, TMVR presents unique challenges because of the complex and highly variable anatomy of the mitral valve [4]. Compared with the relatively symmetrical aortic valve, the mitral valve presents a greater challenge due to its D-shaped, saddle-like annulus, asymmetric anterior and posterior leaflets, and sub-valvular apparatus, including the chordae tendineae and papillary muscles [13, 14]. These anatomical features are not only complex but also variable between patients and are often altered by pathological processes such as mitral annular calcification or leaflet prolapse [15].

To ensure procedural success, TMVR devices must achieve secure anchoring, effective sealing, and unobstructed left ventricular outflow [4]. However, current preclinical design paradigms are hampered by insufficient knowledge of population-level mitral valve anatomical variability [7]. Traditional clinical methods of mitral valve assessment, such as 2D echocardiography, real-time 3D transoesophageal echocardiography (TEE), and CT imaging, offer partial views of mitral anatomy but are limited in resolution relative to key structures such as the mitral leaflets and the papillary posts. 2D echocardiography captures dynamic leaflet motion with high temporal resolution (25–40 Hz) [16] but insufficient spatial resolution (~0.6–3 mm in only two dimensions) [16] to resolve thin leaflets (~0.5–2 mm thick), chordae tendineae (~0.3–0.6 mm), or papillary insertions [13, 17–20]. 3D TEE improves spatial detail (~0.6–1.5 mm in three spatial dimensions) of the annulus and leaflet coaptation but has reduced temporal resolution (~20–40 Hz) and can miss fast sub-valvular motion [21]. CT provides excellent spatial resolution (~0.3–0.6 mm) for delineating the full mitral apparatus but poor temporal resolution (~4–13 Hz), which limits functional insight [22]. These constraints hinder population-level comparisons of 3D anatomy and simulations [23], leading most computational models to rely on idealised or single-patient geometries that fail to capture the diversity of mitral valve morphology observed clinically [24–27].

Several studies have attempted to characterise mitral valve geometry via image-based modelling, often focussing on either isolated cases or small cohorts or reducing the shape to a handful of landmark‑based metrics. These numerous efforts have provided insights into specific anatomical features. For example, Lee et al. 2013 used real-time 3D TEE to quantify annular and leaflet morphology in patients with mitral valve prolapse [23]. Additionally, Oliveira et al. (2020) reviewed geometric findings from cadaveric and imaging data to define average mitral valve anatomy [13]. Despite these advances, approaches that quantitatively capture global mitral valve shape variation in a scalable manner remain relatively limited. These studies are constrained by geometric simplifications, univariate shape analysis [23] or predefined shape parametrisations—such as reliance on ideal ratios such as those proposed by Oliveira et al. 2020—which do not account for the true heterogeneity of mitral valve anatomies observed in clinical populations [28]. These approaches typically assume idealised relationships between annular proportions [13], smooth curvature [13], or symmetrical leaflet geometry, neglecting outlier shapes and subtle but potentially clinically significant deviations that could be introduced by age, pathology, or anatomical diversity. As a result, such models may not adequately represent the anatomical extremes that may challenge device design or procedural outcomes.

To better characterise population-level variability in mitral valve anatomy, this study employs statistical shape modelling (SSM) as a data-driven framework that captures intrinsic morphological variation without reliance on predefined geometric templates [29]. By creating 3D representations of patient anatomies and analysing shape variation using principal component analysis (PCA), the SSM produces a compact set of mathematical descriptors, or principal components, that collectively capture dominant modes of mitral valve shape change. Modes represent correlated variation of geometric features, including annular size, saddle height, leaflet curvature, and commissural width. These descriptors can be used to better understand the anatomical variables present in the population. They can also be used to generate synthetic or representative geometries that accurately span the observed anatomical variation [30, 31]. This capability enables the creation of virtual patient cohorts that reflect real-world diversity and are suitable for in silico device testing, risk stratification, and/or personalised treatment planning.

In cardiovascular applications, the SSM has been used to investigate the geometry of the aortic root [30, 32], left ventricle [33, 34], and aortic valve [35, 36]. However, its application to the mitral valve remains limited. Existing studies have focussed primarily on the annulus, used simplified geometric descriptors [35], or analysed paediatric rather than adult populations [37]. For example, Dabiri et al. (2021) performed PCA on mitral valve geometries generated by morphing a template model to fit six homologous landmark points extracted from the echocardiographic data of 29 patients. However, relying on only six key points to define valve shape introduces a simplification, potentially omitting important anatomical details and limiting the model’s ability to capture the full complexity of mitral valve morphology. Amin et al. 2024 demonstrated the potential of SSM to capture developmental variation in the mitral annulus, yet their model excluded leaflets and other sub-valvular components, structures critical to TMVR planning and simulation [37]. Other researchers have conducted PCA on linear measurements of mitral valve shape to generate virtual patient cohorts of the mitral valve [35]. However, this analysis does not capture the elements of shape that cannot be captured with standardised linear measurements, such as 3D annular nonplanarity, surface curvature, or regional thickness variation. Thus, a comprehensive, adult-focussed SSM of the entire mitral valve, incorporating leaflets and surrounding anatomy, remains an unmet need.

To address this gap, we construct a statistical shape model of the mitral valve using prospectively gated contrast-enhanced coronary CT angiography (CCTA) data from a clinical cohort. CCTA is a non-invasive imaging modality that combines CT scanning with intravenous contrast injection to visualise coronary obstructions in high detail. CCTA provides volumetric data in four dimensions that are well suited for 3D reconstruction of whole cardia and heart valves (~0.3–0.6 mm in plane, ~ 0.5–0.75 mm slice thickness, and ~ 75–250 ms temporal resolution) [38]. While real-time 3D TEE offers excellent temporal resolution for the dynamic assessment of mitral valve function, it is limited by lower spatial resolution, a restricted field of view, and variability due to operator dependence [39, 40]. In contrast, CCTA provides high-resolution, standardised, volumetric data that enable precise visualisation of the mitral annulus, leaflets, and sub-valvular structures in predefined frames of the cardiac cycle using prospective electrocardiography (ECG) gating [38, 40, 41]. These qualities make CCTA particularly well suited for creating anatomically accurate, population-wide geometries of mitral anatomy. By leveraging CCTA in a reproducible computational pipeline, we aim to capture the full spectrum of mitral valve morphology that will be beneficial for device simulation, preprocedural planning, and could help inform the design of next-generation TMVR technologies.

## Methods

### Dataset and Segmentation

This study utilised prospectively gated contrast-enhanced coronary CT angiography (CCTA) scans from 72 adults sourced from the orCaScore dataset—a publicly available dataset developed to support research in automated coronary artery analysis [42]. DICOM scans were sourced through the public repository portal (Synapse), with permission granted by the data owner. Full details of image acquisition and patient population can be found in their publication (full information can be found in Wolterink et al., 2016). Briefly, patients were imaged at four academic hospitals across Belgium and the Netherlands (Antwerp University Hospital; Radboud University Nijmegen Medical Centre, University Medical Centre Groningen, University Medical Centre Utrecht). ECG-triggering was used to prospectively synchronise scan acquisition to the diastolic rest period at 70% (GE, Siemens), 75% (Toshiba), or 78% (Philips) of the R–R interval. It is important to note that, as such, the statistical shape model reflects inter-subject anatomical variability only and does not incorporate within-cycle (temporal) deformation [43]. The set consists of an equal number of male and female patients sampled from four cardiovascular disease risk categories (full information can be found in Wolterink et al., 2016). In brief, patients were free from anatomical abnormalities, intracoronary stents, and metal implants, and all included scans were free from severe motion artefacts and high noise levels. No other scan-matched demographic data were available for these patients. The standardised prospective ECG-gated CCTA images provided detailed structural data suitable for 3D anatomical modelling of the mitral valve.

Initial segmentation was performed using the Simpleware Cardiac Auto Segmentation (AS) tool in Simpleware™ Medical software (Version X-2025.06; Synopsys, Inc., Sunnyvale, USA), which automatically delineates major cardiac chambers from the CCTA volumes. A custom postprocessing script was then applied to extract the mitral annulus and the anterior and posterior leaflets from these chamber segmentations. This process ensured consistent and anatomically guided annular identification across the dataset.

**Fig. 1:**
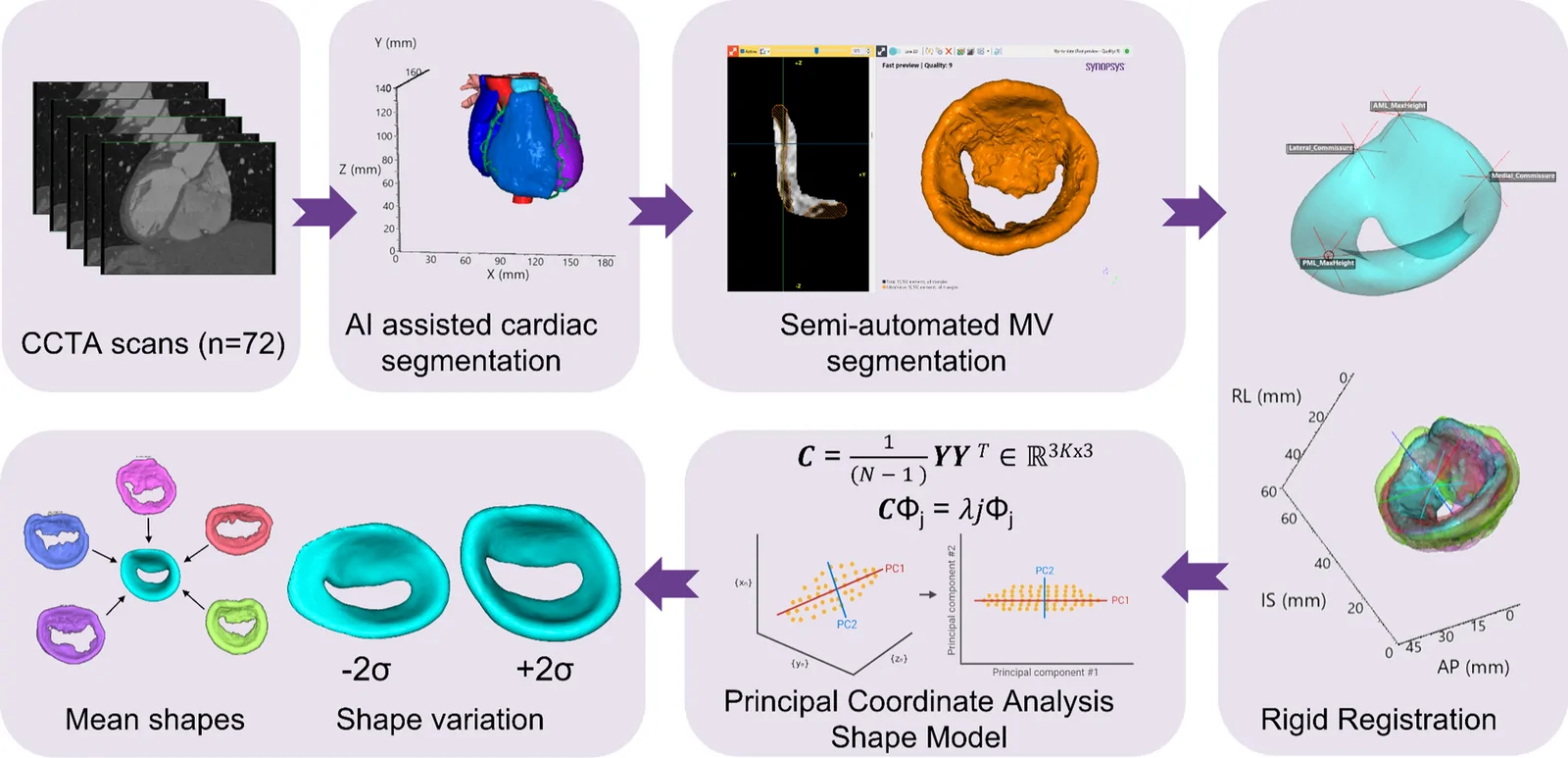
Workflow overview from CTA segmentation to statistical shape model (SSM)

All the segmentations were manually reviewed, and minor defects, such as holes, segmentation noise, or edge artefacts, were corrected via the Simpleware software’s region-growing and mesh editing tools. This semiautomated workflow (Fig. 1) enabled the efficient reconstruction of mitral valve structures, specifically the annulus and anterior and posterior leaflets, across the cohort. A standardised apical view of all 72 segmentations can be viewed in Supplementary Fig. S1.

### Statistical Shape Modelling

SSM pre-processing and construction was conducted using the Simpleware SSM Toolkit and Simpleware Scripting tools (Version X-2025.06; Synopsys, Inc., Sunnyvale, USA) to capture the variability in mitral valve geometry across the population of 72 adults. Unlike approaches based on exclusively manual landmark assignment and correspondence, this pipeline uses full 3D segmentation representations as the starting point, allowing for high-resolution shape analysis without manually defined anatomical points.

All segmentations were pre-processed to ensure consistent topology. To limit the impact of rotational and translational variations in scan acquisition, a registration algorithm was applied using four anatomical landmarks that were manually placed on each segmentation (Fig. 1). Four anatomical landmarks were manually identified on the mitral annulus to guide rigid registration. Two landmarks were defined along the anterior–posterior axis of the annulus: (1) the most superior point of the annulus overlying the midpoint of the anterior mitral leaflet (A2 scallop), and (2) the most superior point of the annulus overlying the midpoint of the posterior mitral leaflet (P2 scallop). The remaining two landmarks were placed at the annular hinge points corresponding to the anteromedial commissure (A1–P1 junction) and the posterolateral commissure (A3–P3 junction) [44]. These landmarks were then used as points for a best-fit rigid transformation to align two surface representations by iteratively minimising the distance between corresponding points, effectively removing differences in translation and rotation while preserving scale and geometry.

PCA was then performed on the aligned segmentations. The shape variance is evaluated as a vertex-wise geometric variation relative to the reference geometry. PCA decomposes the variance in these shape vectors into orthogonal principal components (PCs), which represent uncorrelated modes of morphological variation within the population. The mean shape was calculated, and each individual shape was expressed as a linear combination of the mean and the PCs as per Eq.1:

1 $$x_{new} = \overline{x} + \mathop \sum \limits_{j = 1}^{m} \alpha_{j} \sqrt {\lambda_{j} } \nu_{j}$$

where $\overline{x }$ is the mean shape, ${\nu }_{j}$ are the eigenvectors (principal components), ${\lambda }_{j}$ are the eigenvalues (explained variance), and ${\alpha }_{j}$ are the shape parameters sampled across each mode. To explore the full anatomical variation within the dataset, we generated synthetic mitral valve geometries by varying ${\alpha }_{j}$ within a range of ± 2 standard deviations (σ) along each of the first three principal components. These synthesised shapes represent a wide morphological envelope that includes both typical and extreme anatomical presentations.

To characterise the anatomical variability in the sample population, key geometric dimensions of the mitral valve were assessed as a function of variation along the shape modes (PCs). These measurements were mapped automatically from the reference model to each of the input models using the Simpleware measurement tools and the Simpleware SSM Toolkit (Version X-2025.06). Anterior mitral leaflet (AML) length was defined as the maximum superior–inferior distance between the free edge of the anterior leaflet and its annular insertion. Posterior mitral leaflet (PML) length was defined analogously as the maximum superior–inferior distance between the free edge of the posterior leaflet and its attachment to the mitral annulus.

The anterior–posterior (A–P) annular dimension was defined as the linear distance between the annular midpoint corresponding to the A2 scallop and the opposing annular midpoint corresponding to the P2 scallop. The intercommissural (IC) distance was defined as the distance between the anteromedial and posterolateral commissures of the mitral valve.

A valve height metric was defined to characterise anterior leaflet elevation relative to the commissural reference, defined as the vertical distance between the most superior point of the anterior mitral leaflet and the plane defined by the anteromedial and posterolateral commissures.

To facilitate automated measurement mapping, the 3D points representing the measurements listed above were extracted from the reference image and constrained to a specific anatomical region defined by a binary mask. This same anatomical region was identified in a second target image using an equivalent mask. The spatial relationship defined by the statistical shape model, and constrained by the reference and target masks, was used to determine how each point from the reference should be repositioned within the target anatomy, preserving anatomical correspondence. Once the points were repositioned, they were converted from the reference’s global coordinate system into the target’s image coordinate system, ensuring that they were correctly aligned and interpretable in the context of the target image.

### Model Assessment

Model assessment was evaluated using three complementary assessments: proxy specificity (reconstruction fidelity), generalisability, and model stability.

Reconstruction fidelity was quantified by reconstructing each input shape using the PCs and calculating the Dice–Sørensen similarity coefficient (DICE score) between the reconstructed geometry and the original geometry. The mean DICE score from all 72 regenerated inputs was used as a proxy specificity metric of the whole SSM. This has been done in comparable cardiac SSMs [43, 45]. For example, Carminati et al. (2018) reported a DSC > 0.84 when reconstructing LV endocardial and epicardial surfaces on held‑out CMR cases [45].

Generalisability (a model performance metric) was assessed using a leave-one-out approach, in which each excluded shape was reconstructed using an SSM built from the remaining samples, and the reconstruction error was quantified as the root-mean-square Euclidean distance (RMSE) between corresponding points, consistent with the definition of model generalisation described by Audenaert et al., 2019 [46]

SSM model stability was assessed using the Mahalanobis distance. For a series of candidate model dimensionalities (70, 65, 60, 55, 50, 45, 40, 35, 30, 25, and 20 PCs), we regenerated all input shapes into the corresponding truncated PC space. For each configuration, we then computed the Mahalanobis distance of every shape to the model mean in PC space and recorded how many regenerated inputs exceeded the 95% Mahalanobis threshold. This allowed us to detect when regenerated anatomies become statistically inconsistent with the full PCA distribution represented by the model. The number of regenerated inputs that exceeded the Mahalanobis 95% threshold decreased systematically as more components were included and reached zero at 50 PCs and above, indicating that the model stabilised at this dimensionality (Supplementary Figure 2).

## Results

**Fig. 2:**
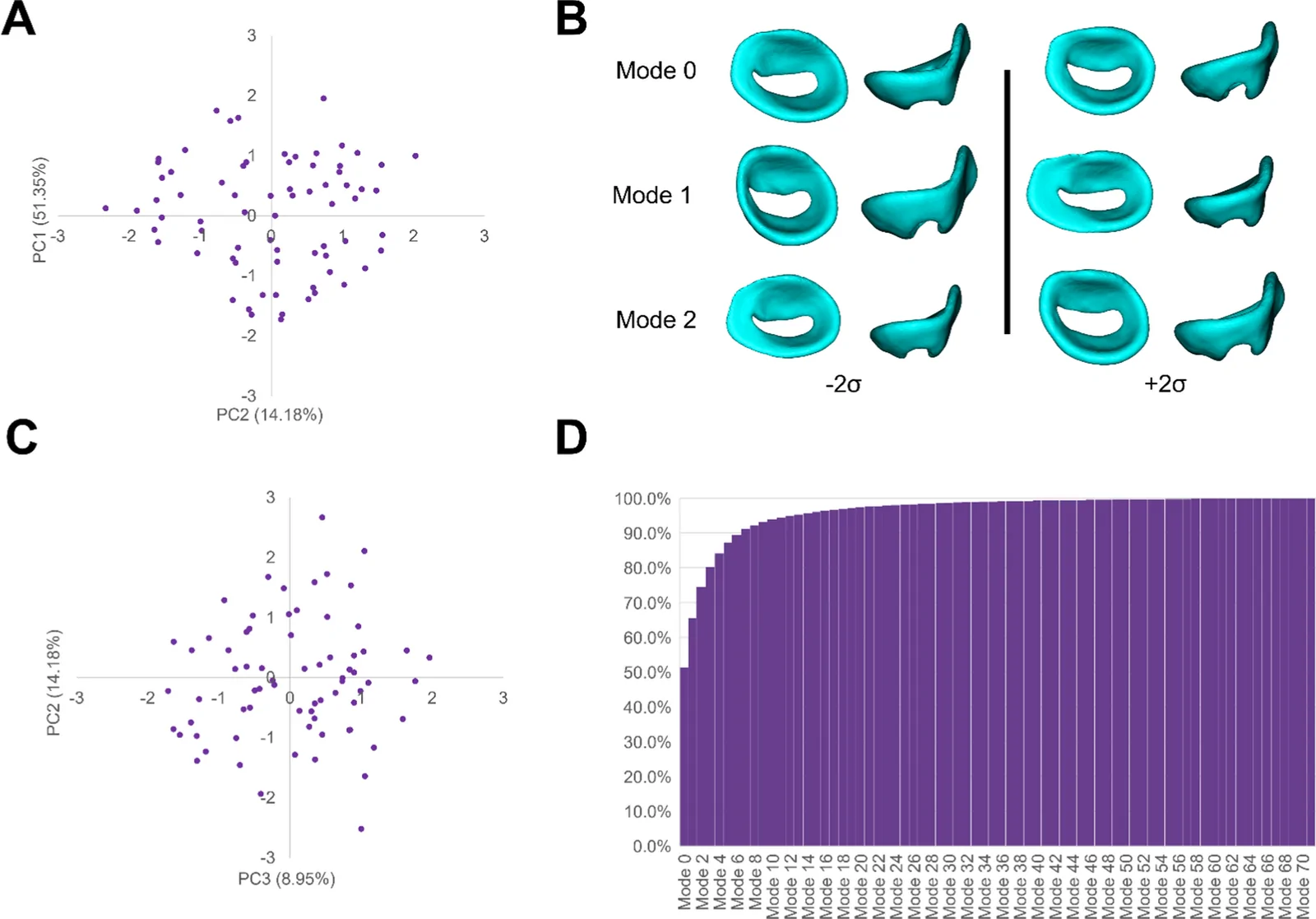
**A** Principal Component (PC) 1 vs. PC2 scores for all the samples in the dataset. **B**  ± 2 standard deviation geometries along the first three modes of variation. **C** PC2 vs. PC3 scores for all the samples in the dataset. **D** Compactness (cumulative variance) plot of the shape mode

PCA was used to characterise the dominant modes of geometric variation in the mitral valve shapes. The first four principal components explained a cumulative 84.12% of the total variance, with PC1 (Mode 0) accounting for 51.36%, PC2 (Mode 1) accounting for 14.18%, and PC3 (Mode 2) accounting for 8.95% (Fig. 2A, 2C). The first twelve principal components accounted for > 90% of the total variance in mitral valve shape (Fig. 2D).

The cumulative variance plot (Fig. 2D) further demonstrated that the first three PCs captured most of the shape variability in the dataset. Beyond the third component, additional PCs contributed only marginal increases in explained variance, indicating that most of the meaningful geometric information is contained within the leading modes.

**Fig. 3.**
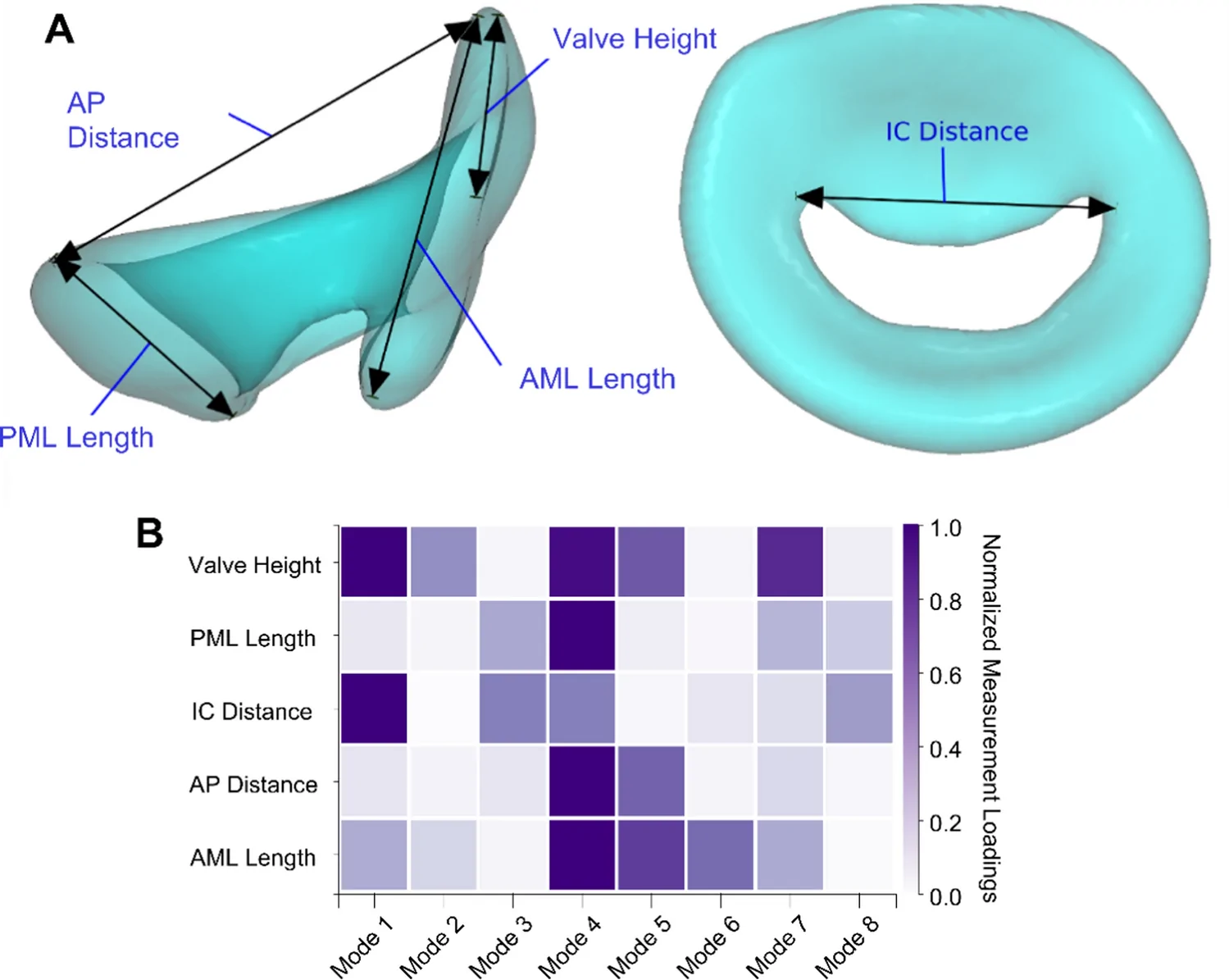
**A** Geometric representation of the mapped measurements of the mitral valve, *AP* anterior posterior distance, *AML* anterior mitral leaflet length, *PML* posterior mitral leaflet length, *IC* intercommissural distance, and valve height. **B** Heatmap of the mapped measurements loadings relative to the shape modes

To interpret the anatomical significance of the principal components, representative mitral valve geometries were reconstructed at ± 2 standard deviations along each of the first three PCs (Fig. 2B). Additional interpretation and inspection were completed by looking at the 3D geometries of the first three PCs ± 2 standard deviations (Supplementary Data 1). To quantify how these modes relate to clinically interpretable geometric features, five linear measurements—AP diameter, AML length, PML length, intercommissural (IC) distance, and valve height—were automatically mapped onto all training shapes and regressed onto the corresponding principal component scores. The resulting coefficients were normalised to a − 1 to + 1 scale for visualisation in Fig. 3B, where they represent the relative loading of each measurement on each mode. These loadings indicate the degree to which each anatomical measurement contributes to variation along a principal component; however, they are not Pearson correlation coefficients and should not be interpreted as implying perfect statistical correlation.

Based on these loadings and the ± 2 SD reconstructions, PC1 (Mode 0) primarily reflects overall valve size, with its strongest contributions from IC distance and valve height. PC2 (Mode 1) influences multiple geometric features simultaneously, with the strongest associations being AML length and valve height, consistent with the combined shape variations observed in the reconstructed geometries. PC3 (Mode 2) is most strongly associated with IC distance, with additional contributions from AP diameter and PML length, corresponding to variation in the medial–lateral position of the orifice and the balance of leaflet lengths.

Together, these three modes capture the dominant axes of mitral valve shape variation observed in the cohort. PC1 describes uniform scaling of the valve and leaflets, PC2 reflects combined changes in annular shape and leaflet dimensions, and PC3 represents variation in commissural width and AP-oriented leaflet geometry. The agreement between the linear-measurement loadings in Fig. 3B and the geometric differences illustrated by the ± 2 SD reconstructions in Fig. 2 provides a consistent and interpretable basis for understanding the principal components.

PC1, PC2, PC4, and PC5 reflected coordinated changes in posterior leaflet length and the three-dimensional contour of the posterior annulus (Fig. 3B), producing geometric patterns that appear to maintain coaptation area even as annular shape varies. PC3 was associated with reductions in commissural distance and curvature (Figs. 2B and 3B), a pattern consistent with subtle shifts in the effective alignment of the leaflets and sub-valvular apparatus, similar in appearance to variations in papillary muscle orientation that influence leaflet meeting lines. Together, these modes illustrate how normal variation in annular and sub-valvular geometry can support robust leaflet coaptation across a physiologically relevant range of shapes.

The AML length ranged from 23.5 to 36.8 mm, whereas the PML length varied from 13.2 to 25.3 mm, reflecting substantial interindividual variation in leaflet morphology. The valve height, defined as the anterior leaflet elevation relative to the commissural plane, spanned 10.6 to 19.2 mm across the cohort. The IC distance ranged from 27.9 to 38.1 mm, and the AP distance varied from 31.7 to 44.6 mm, indicating a wide spectrum of annular dimensions. These anatomical differences form the basis of the shape variation explored through PCA.

To evaluate the reconstruction fidelity (proxy specificity) of the shape model, input geometries were regenerated and compared to their originals via the Dice similarity score or Sørensen–Dice index. In this case, 61 regenerated shapes achieved Dice similarity scores above 70%, and 11 fell below this threshold, with a mean DSC of 76.71 ± 13.28%. These discrepancies were not attributable to segmentation errors or anatomical anomalies, suggesting limitations in the model’s ability to fully reconstruct certain shapes. This reflects a known limitation of PCA-based statistical shape models: although effective at capturing dominant modes of variation, PCA assumes linearity and Gaussian-distributed shape variation [47, 48]. As a result, complex or nonlinear anatomical features may be underrepresented, particularly when shapes that lie near the boundaries of the shape space are reconstructed. Moreover, as this model represents a volumetric SSM, the corresponding Dice coefficient penalises even slight volumetric mismatches.

Model generalisability was evaluated using a leave-one-out analysis on all 72 specimens, where each held-out shape was reconstructed using a model built without that specimen. This yielded a mean reconstruction error of 0.969 mm, indicating that the model was generally able to represent unseen anatomies with good fidelity.

Together, these tests show that the shape model captures the principal geometric characteristics of the mitral valve while exhibiting robust performance when representing both training and unseen cases.

## Discussion

This study presents a comprehensive SSM of the adult human mitral valve derived from standard CCTA data in a cohort of 72 adults. By leveraging a semiautomated segmentation pipeline and full 3D surface analysis, we provide a detailed map of morphological variation across the mitral annulus and leaflets, offering valuable insights for interventional device development, preprocedural planning, and virtual patient generation.

Although this study excludes pathological mitral conditions, establishing a detailed statistical description of normal 3D morphology is an essential precursor to modelling pathological variation. Prior work has typically examined the annulus in isolation [37] or used sparse landmark-based valve representations [35], and population-level PCA of the full adult mitral valve—including both leaflets—has not previously been reported. By using CCTA as a consistent imaging modality and analysing a standardised cardiac phase (70, 75, and 78% of the R–R interval), the present model isolates inter-subject anatomical variability from imaging-related differences. This provides a necessary baseline against which pathological formations can be interpreted in future studies incorporating prolapsed, regurgitant, or calcified anatomies.

The reconstruction fidelity and generalisability assessments together indicate that the model reliably captures the dominant patterns of mitral valve morphology, with modest error in both input reconstructions and held-out shapes. Our results support the suitability of the statistical shape model as a foundation for population-based anatomical analysis and downstream simulation applications, though future analysis seeks to recreate this pipeline on a bigger dataset to establish model fidelity and improve generalisability. To compare the performance of our model to existing literature, Amin et al. 2024 [37] modelled the dynamic paediatric mitral annulus using PCA but did not report explicit proxy specificity or generalisability metrics, instead focussing on explained variance and qualitative mode interpretation. Dabiri et al. 2021 [35] constructed a mitral valve atlas from six echocardiographic landmarks and primarily validated it via AI prediction performance rather than quantitative shape-reconstruction tests, so—unlike this work—they did not provide model performance metrics that could be used to compared with our DICE reconstruction fidelity and leave-one-out generalisability assessments. Nonetheless, the levels of reconstruction fidelity and generalisability we observed are consistent with those reported for statistical shape models of other cardiac structures [30, 32–34, 36, 43, 45, 49, 50], suggesting that the present model performs within the expected range for high-resolution cardiovascular SSMs.

Our findings confirm that the dominant modes of shape variation—particularly PC1 (size and anterior‒posterior dilation), PC2 (posterior leaflet morphology and commissural distance), and PC3 (orifice asymmetry and leaflet alignment)—represent key axes of anatomical diversity in the adult mitral valve. These conclusions are consistent with prior anatomical and imaging studies describing natural interindividual variability in mitral valve geometry. For example, variation in annular size and anterior‒posterior length (PC1) reflects morphometric differences observed in cadaveric dissections and CT-based analyses of annular shape [13, 14]. Similarly, PC2 corresponds to differences in posterior leaflet length and commissural span, features captured in echocardiographic and imaging studies focussed on defining typical leaflet dimensions [13, 23]. PC3 captures orifice asymmetry and annular torsion, consistent with the known saddle-shaped, nonplanar geometry of the native mitral annulus described in both in vivo and ex vivo studies [35, 37]. The coherence of our results with the findings of previous imaging and dissection studies supports this model’s anatomical validity and its capacity to describe the natural spectrum of mitral valve morphology.

The first four principal components accounted for 84% of the total shape variance, comparable to previous shape-modelling studies in cardiovascular anatomy [34, 36]. Unlike models that rely on simplified measurements or geometric templates, our approach captures complex, multidimensional variability by using full 3D surface representations of the mitral valve. This preserves subtle yet clinically meaningful features—including leaflet curvature, annular nonplanarity, and orifice asymmetry—that are often missed by traditional linear metrics [35] , even when assessing a static frame [51]. Rather than reducing the use of the mitral valve to ideal anatomical templates, this model provides a compact but comprehensive representation of 3D shape diversity in the adult population.

This type of small, yet anatomically rich model may be particularly relevant in the context of TMVR, where anatomical complexity poses challenges to device performance [28]. Unlike the aortic valve, the mitral valve displays high geometric variability, which affects device sizing, anchoring, and interaction with the sub-valvular apparatus [4]. Many current planning workflows rely on idealised or limited-case geometries that may not reflect the diversity encountered in real-world patients [5–8, 52–55]. In contrast, the shape model developed here enables the generation of representative and edge-case anatomies that could better inform device selection and procedural risk [56]. However, the presence of reconstruction error highlights an important consideration for downstream application of the model [57]. While the SSM captures a wide spectrum of mitral valve morphologies, localised geometric inaccuracies may arise in anatomically complex regions, which could influence device–tissue interaction in simulation-based planning. This underscores the need for careful interpretation of regenerated geometries in high-risk regions. Future work may address these reconstruction artefacts by extending the modelling framework to jointly capture mitral–aortic valve anatomy, enabling more consistent representation of annular geometry in the aortic–mitral continuity without imposing explicit geometric constraints.

The anatomical variety found in this study population also reaffirms key anatomical considerations for existing TMVR systems such as Abbott Tendyne and Edwards SAPIEN 3, in keeping with conclusions from human cadaver-based dissection studies [20] . The Tendyne valve, which is designed to conform to a wide range of native annular shapes with a D-shaped atrial cuff and apical tethering [10], may still face difficulties in anatomies with very long posterior leaflets—features captured in PC2, as this may correspond to increased leaflet mobility. These anatomical characteristics could affect tethering behaviour and increase the risk of sealing gaps or paravalvular leak. Similarly, while the SAPIEN 3 valve is balloon-expandable, circular in design, and incorporates a docking system to accommodate the D-shaped mitral annulus [58], anatomies with pronounced asymmetry or nonplanarity—as captured by PC3—may still pose challenges for optimal seating and sealing. These observations are made with the caveat that dominant shape modes identified here represent those of non-pathological mitral anatomy and may not reflect a pathological population. Nevertheless, they highlight the ongoing need to tailor device design to the complex and variable geometry of the mitral annulus.

The range of leaflet lengths and annular dimensions observed in this dataset exceeds what current sizing protocols typically account for [59] but does not exceed values reported in clinical literature. A data-driven approach such as the one presented here can inform more adaptive design strategies for next-generation devices—whether through more flexible anchoring mechanisms, improved sealing cuffs, or optimised frame geometries. These models also offer a pathway towards preoperative classification of patients based on anatomical suitability for specific devices or delivery approaches.

Equally important to physical device design, this model provides a foundation for several forward-looking applications. One is the development of virtual imaging trials, in which synthetic but anatomically realistic mitral valves are used to assess image-based planning tools, segmentation algorithms, or sizing protocols across a diverse range of shapes. These findings support performance evaluation under fully controlled conditions and can supplement early-phase clinical or regulatory studies. By sampling across principal component space, virtual cohorts can be constructed to reflect the true variability in mitral anatomy, improving the generalisability of imaging pipelines and reducing bias towards idealised geometries [50].

This statistical shape model could be incorporated into early-stage in silico evaluation workflows by providing population-representative geometries for preliminary device–anatomy interaction studies, consistent with V&V40 guidance [60] model contextualisation and risk-appropriate use. In this capacity, the model supports upstream design-space exploration and virtual cohort generation, complementing but not replacing higher-fidelity sub-models that inform later phases of device development.

We plan to use this model to generate a population of virtual patient models for finite element simulation of the mitral valve. By assigning realistic material properties and boundary conditions to synthetic geometries, we intend to simulate deformation, coaptation, and stress distribution under physiologic loading. It is important to note that CCTA-derived leaflet surfaces represent the externally reconstructed leaflet geometry rather than true histological thickness. As a result, the SSM provides a segmentation-based proxy leaflet thickness. Nevertheless, using the reanimated morpho-space of the mitral valve, we aim to find clearer connections between the specificities of mitral valve anatomy in this population and leaflet stress. In the future, this model paradigm could also enable predictive modelling of device–tissue interactions to help identify anatomies at risk for complications such as paravalvular leak or LVOT obstruction and to support the refinement of device features before physical prototyping.

While this study offers a strong foundation, several limitations should be acknowledged.

Firstly, the orCaScore dataset contains only anatomically healthy mitral valves, limiting direct applicability to populations with mitral pathology. Expanding the dataset to include diseased valves—such as those affected by regurgitation, prolapse, or annular calcification—would enhance the model’s utility.

A further limitation concerns the reconstruction fidelity of the shape model. While the average Dice similarity score was acceptable (76.71%), multiple anatomies exhibited lower values. These discrepancies were not attributable to segmentation quality or anatomical outliers but we believe instead reflect the well-recognised constraints of PCA-based statistical shape models [61]. As PCA assumes linearity and Gaussian variation, it may not fully capture highly localised or nonlinear geometric differences—particularly in thin, complex regions such as leaflet free edges or commissural curvature. The volumetric Dice coefficient is also a stringent metric for this application, as even small surface deviations in leaflet geometry can lead to disproportionately reduced segmentation overlap. Although PCA provides a compact and interpretable representation of population-level shape variation, it is limited to modelling linear modes of deformation and may therefore overlook subtle nonlinear features of leaflet curvature or annular torsion. More advanced nonlinear shape-modelling approaches (e.g. kernel methods or neural-network–based latent spaces) could in principle address these behaviours, but they require substantially larger training cohorts and more complex regularisation than was feasible with the present dataset. Future work will explore these techniques as larger, multi-centre 4D imaging datasets become available, and it becomes possible to explore nonlinear approaches that may better represent anatomies at the extremes of the shape space and improve reconstruction fidelity.

Upon more thorough visual inspection, a proportion of regenerated input shapes exhibited non-physiological characteristics, highlighting a limitation of the current SSM that should be considered in downstream applications. Namely, qualitative assessment of the regenerated inputs demonstrated a common non-physiological error—the generation of geometries with annular curves exhibiting departure from the original annular contour at the aortic commissure. This was investigated further by regenerating all input shapes using PC truncated SSMs (70, 65, 60, 55, 50, 45, 40, 35, 30, 25, and 20 PCs). For each configuration, qualitative assessment of the annular curves was conducted to determine the number of regenerated inputs that failed to meet the assessment criteria. Specifically, regenerated shapes were classified as non-physiological when the mitral annulus exhibited a localised geometric artefact near the aortic (anteromedial) commissure, characterised by abnormal curvature and loss of a well-defined annular contour relative to the original segmentation. Results (Supplementary Figure 3) demonstrate that increasing the number of modes used to regenerate inputs does not show a clear or systematic relationship with the number of non-physiological inputs generated. Despite this limitation, the SSM remains consistent with state-of-the-art approaches for modelling complex valvular anatomy [62], capturing the dominant modes of physiological variation while highlighting the need for additional anatomical constraints in future refinements.

Another important limitation of this study is the use of single-phase, ECG-gated CCTA data, which restricts the model to static anatomical information. Although these images provide excellent spatial resolution for detailed reconstruction of the mitral annulus and leaflets, they do not capture the dynamic behaviour of the valve throughout the cardiac cycle. As a result, temporal changes in annular nonplanarity, leaflet curvature, coaptation, and sub-valvular motion cannot be represented within the current shape space and/or compared across individuals. Dynamic (4D) CCTA or real-time 3D TEE would enable modelling of phasic deformation patterns and permit statistical analysis of valve kinematics, which are increasingly recognised as important determinants of leaflet stress, regurgitation mechanisms, and device–tissue interaction. Incorporating such datasets into future work would allow construction of a spatiotemporal SSM capable of capturing both geometric and functional variability, thereby enhancing the relevance of virtual patient cohorts for simulation-based planning and TMVR device evaluation.

Finally, the lack of scan-matched patient demographic data precludes analysis of how age, sex, or body size may contribute to variation.

Despite these limitations, this work represents the first statistical shape model of the adult human mitral valve to date. It provides a reproducible and scalable framework to support device design, virtual cohort generation, and simulation-based testing. As TMVR technologies continue to evolve, anatomically grounded modelling approaches will be critical for improving performance, expanding eligibility, and personalising care across diverse patient populations.

## Conclusion

Our CCTA-based statistical shape model captures the majority of shape variation in the normal adult mitral valve geometry in our population in four dominant modes, offering an anatomically rich yet compact framework for TMVR design, virtual imaging trials, and future finite element simulations.

## Supplementary Information

Below is the link to the electronic supplementary material.

Supplementary file1 (JPG 998 KB)

Supplementary file2 (PNG 49 KB)

Supplementary file3 (PNG 41 KB)

Supplementary file4 (DOCX 15 KB)

## Data Availability

The underlying computed tomography angiography (CTA) dataset used in this study is publicly available as part of the orCaScore database [42]. Although the compiled statistical shape model cannot be redistributed due to licensing constraints of the commercial SSM software, the full set of mitral valve segmentations used to build the model is available from the corresponding author upon reasonable request. Together with the detailed description of the full processing pipeline provided in this manuscript, these segmentations enable other researchers to reconstruct comparable statistical shape models using their own preferred SSM frameworks.
