# Supplementary material for: Morphological Variation in the Human Mitral Valve Using Statistical Shape Modelling
Source: Ann Biomed Eng. 2026 Mar 19;54(10):3465–76. doi: 10.1007/s10439-026-04060-6 (PMC13649912; doi:10.1007/s10439-026-04060-6)
Supplement: Supplementary file 1 [file 10439_2026_4060_MOESM4_ESM.docx]

# Supplementary Legends

Supplementary Figure 1: Original mitral valve segmentations derived from OrcaScore coronary computed tomography angiography (CCTA) dataset and used as input for statistical shape model (SSM) construction. All segmentations are shown prior to alignment, correspondence establishment, and model generation.

Supplementary Figure 2: Statistical shape model (SSM) stability analysis based on Mahalanobis distance. Input shapes were reconstructed using truncated PCA-based SSMs with varying numbers of retained modes, and the number of reconstructions exceeding the 95% Mahalanobis threshold was recorded. Exceedances decrease with increasing model dimensionality and reach zero at 50 PCs, indicating stabilisation of the SSM beyond this dimensionality.

Supplementary Figure 3: Statistical shape model (SSM) of mitral valve stability analysis based on regenerated input assessment. Inputs were reconstructed using truncated PCA-based SSMs with varying numbers of retained modes, and the number of reconstructions displaying annular distortion at aortic commissure were recorded (qualitative assessment).

Supplementary Data 1: Rotatable surface geometries of the mean mitral valve shape and the ±2 standard deviation (SD) variations along the first two principal components from a mitral valve statistical shape model (SSM). These models illustrate the dominant modes of anatomical variation captured by the SSM.

Available for reviewers to view temporarily by link – https://rcsicampus-my.sharepoint.com/:p:/g/personal/hyabmehariabraha_rcsi_com/IQALx8tQJPwhRbuVxHK-D8JnAbkhb-DJxFFMNmxKeCirKIQ?email=hyabmehariabraha%40rcsi.ie&e=pNXSrs
